# Supplementary material for: Synthesis, Molecular Simulation, DFT, and Kinetic Study of Imidazotriazole-Based Thiazolidinone as Dual Inhibitor of Acetylcholinesterase and Butyrylcholinesterase Enzymes
Source: Pharmaceuticals (Basel). 2025 Mar 15;18(3):415. doi: 10.3390/ph18030415 (PMC11944621; doi:10.3390/ph18030415)
Supplement: Supplementary file 1 [file pharmaceuticals-18-00415-s001.zip › pharmaceuticals-3500250-supplementary.pdf]

Synthesis, molecular simulation, DFT and kinetic study of imidazotriazole based thiazolidinone as dual inhibitor of acetylcholinesterase and butyrylcholinesterase enzymes

M.M. Khowdiary[1], Shoaib Khan[2*], Tayyiaba Iqbal[2], Wajid Rehman[3], Muhammad Bilal Khan[2], Mujaddad Ur Rehman[4], Zanib Fiaz[2], Hakimullah[5]

[1]*Department of Chemistry, Faculty of Applied Science, University College-Al Leith, University of Umm Al-Qura, Makkah 21955, Saudi Arabia.*
[2]*Department of Chemistry, Abbottabad University of Science and Technology, Abbottabad, 22500, Pakistan.*
[3]*Department of Chemistry, Hazara Univeristy Mansehra, 21120, Pakistan.*
[4]*Department of Microbiology, Abbottabad University of Science and Technology, Abbottabad, 22500, Pakistan.*
[5]*Department of Chemistry, Balochistan University of Information and Technology,Engineering & Management Sciences, Quetta, Pakistan.*

[*]Corresponding author: shoaibkhanswati@gmail.com (S. *Khan*)

Supplementary Information

Graph-S1: Percentage inhibition of analog 3 vs. inhibitor concentration which illustrates the drug-dose relationship against AChE

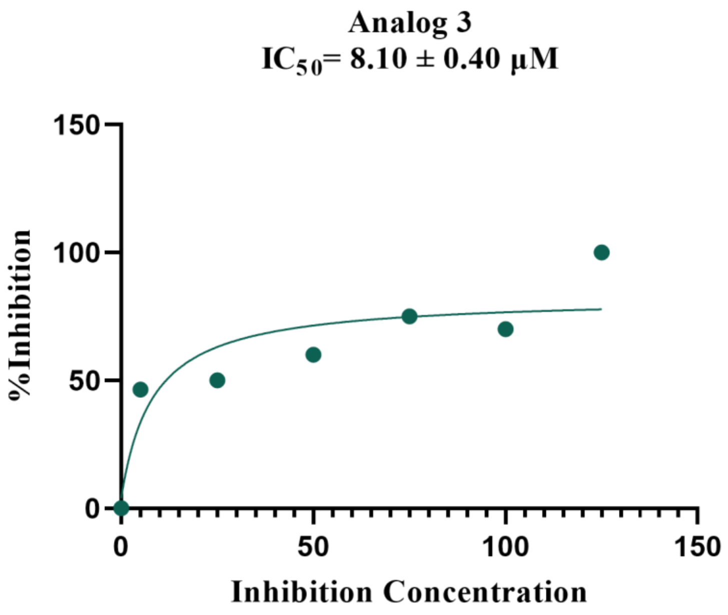

Graph-S2: Percentage inhibition of analog 3 vs. inhibitor concentration which illustrates the drug-dose relationship against BChE

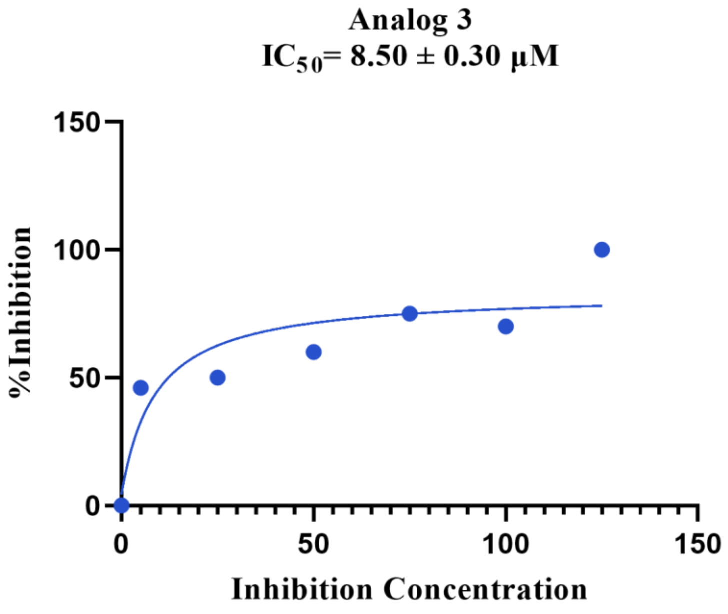

Graph-S3: Percentage inhibition of analog 11 vs. inhibitor concentration which illustrates the drug-dose relationship against AChE

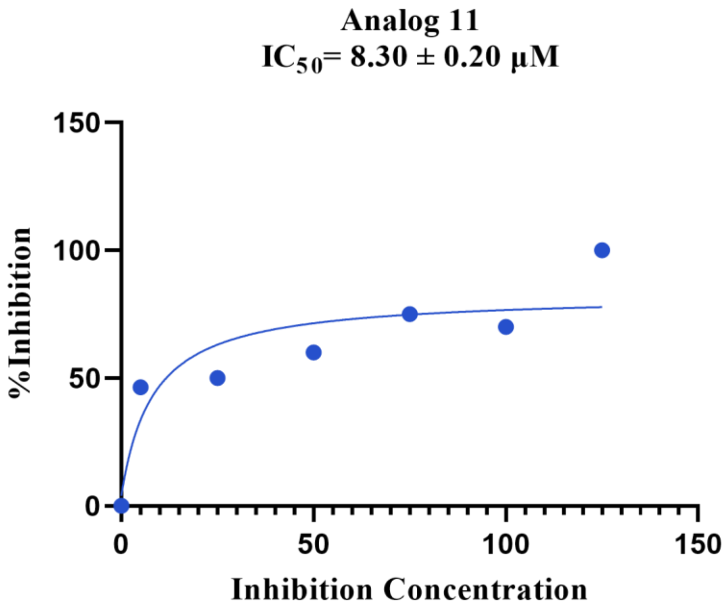

Graph-S4: Percentage inhibition of analog 11 vs. inhibitor concentration which illustrates the drug-dose relationship against BChE

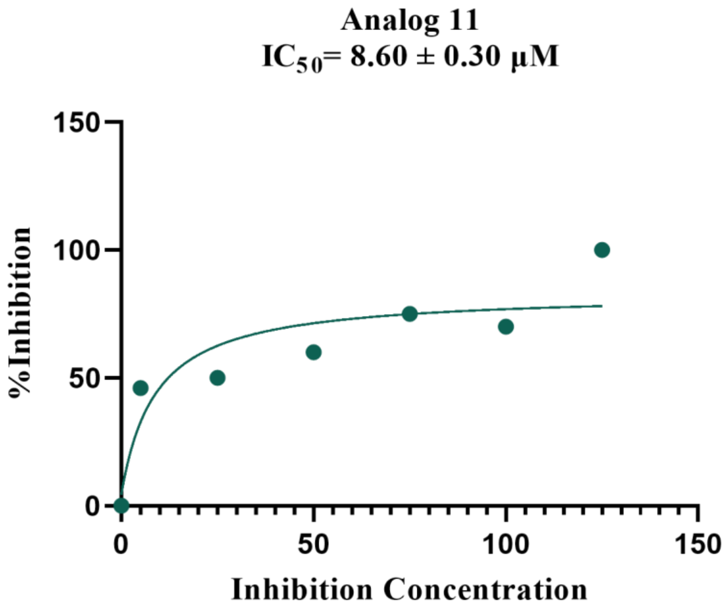

Table-S1: ADMET predictions for analog 10 and its toxilogical profile

Property	Model Name	Predicted Value	Unit
**Absorption**	Water solubility	**-2.892**	Numeric (log mol/L)
**Absorption**	Caco2 permeability	**1.26**	Numeric (log Papp in 10^{-6} cm/s)
**Absorption**	Intestinal absorption (human)	**89.584**	Numeric (% Absorbed)
**Absorption**	Skin Permeability	**-2.735**	Numeric (log Kp)
**Absorption**	P-glycoprotein substrate	**Yes**	Categorical (Yes/No)
**Absorption**	P-glycoprotein I inhibitor	**Yes**	Categorical (Yes/No)
**Absorption**	P-glycoprotein II inhibitor	**Yes**	Categorical (Yes/No)
**Distribution**	VDss (human)	**-0.117**	Numeric (log L/kg)
**Distribution**	Fraction unbound (human)	**0.263**	Numeric (Fu)
**Distribution**	BBB permeability	**-1.613**	Numeric (log BB)

**Distribution**	CNS permeability	**-2.311**	Numeric (log PS)
**Metabolism**	CYP2D6 substrate	**No**	Categorical (Yes/No)
**Metabolism**	CYP3A4 substrate	**No**	Categorical (Yes/No)
**Metabolism**	CYP1A2 inhibitior	**Yes**	Categorical (Yes/No)
**Metabolism**	CYP2C19 inhibitior	**Yes**	Categorical (Yes/No)
**Metabolism**	CYP2C9 inhibitior	**Yes**	Categorical (Yes/No)
**Metabolism**	CYP2D6 inhibitior	**No**	Categorical (Yes/No)
**Metabolism**	CYP3A4 inhibitior	**Yes**	Categorical (Yes/No)
**Excretion**	Total Clearance	**0.353**	Numeric (log ml/min/kg)
**Excretion**	Renal OCT2 substrate	**Yes**	Categorical (Yes/No)
**Toxicity**	AMES toxicity	**Yes**	Categorical (Yes/No)
**Toxicity**	Max. tolerated dose (human)	**0.488**	Numeric (log mg/kg/day)
**Toxicity**	hERG I inhibitor	**No**	Categorical (Yes/No)
**Toxicity**	hERG II inhibitor	**Yes**	Categorical (Yes/No)
**Toxicity**	Oral Rat Acute Toxicity (LD50)	**2.24**	Numeric (mol/kg)
**Toxicity**	Oral Rat Chronic Toxicity (LOAEL)	**0.912**	Numeric (log mg/kg_bw/day)
**Toxicity**	Hepatotoxicity	**Yes**	Categorical (Yes/No)
**Toxicity**	Skin Sensitisation	**No**	Categorical (Yes/No)
**Toxicity**	*T.Pyriformis* toxicity	**0.285**	Numeric (log ug/L)
**Toxicity**	Minnow toxicity	**1.204**	Numeric (log mM)

S.1 Spectral Analysis

1. 3-(4-(7H-imidazo[2,1-c][1,2,4]triazol-3-yl)-2-methoxyphenyl)-2-(4-fluorophenyl)thiazolidin-4-one

^{1}H NMR (600MHz, DMSO-d_6): δ 12.96 (s, 1H, NH-imidazole), 7.73 (d, J = 8.46 Hz, 1H, Ar-H), 7.41 (d, J = 8.40 Hz, 1H, Ar-H), 7.32 (s, 1H, Ar-H), 7.25 (d, J = 8.70 Hz, 2H, Ar-H), 7.18 (d, J = 6.82 Hz, 2H, Ar-H), 7.12 (d, J = 7.26 Hz, 1H, H-imidazole), 7.08 (d, J = 8.99 Hz, 1H, H-imidazole), 6.42 (s, 1H, H-thiazolidinone), 3.95 (s, 2H, H-thiazolidinone), 3.82 (s, 3H, CH_3); ^{13}C NMR (150 MHz, DMSO-d_6): δ 171.1, 161.2, 153.1, 152.7, 152.4, 135.8, 134.6, 130.3, 130.1, 128.4, 127.1, 123.2, 120.1, 115.4, 115.3, 114.3, 111.2, 72.6, 55.6, 33.2; HREI MS:*m/z* calcd for $C_{20}H_{16}FN_5O_2S$ $[M]^+$ 409.44 Found 409.40.

2. 3-(4-(7H-imidazo[2,1-c][1,2,4]triazol-3-yl)-2-methoxyphenyl)-2-(3,5-difluorophenyl)thiazolidin-4-one

^{1}H NMR (600MHz, DMSO-d_6): δ 12.95 (s, 1H, NH-imidazole), 7.76 (d, J = 8.46 Hz, 1H, Ar-H), 7.43 (d, J = 7.38 Hz, 1H, Ar-H), 7.31 (s, 1H, Ar-H), 7.18 (d, J = 7.15 Hz, 1H, H-imidazole), 7.04 (s, 2H, Ar-H), 7.00 (s, 1H, Ar-H), 6.88 (d, J = 7.20 Hz, 1H, H- imidazole), 6.41 (s, 1H, H-thiazolidinone), 3.94 (s, 2H, H-thiazolidinone), 3.81 (s, 3H, CH_3); ^{13}C NMR (150 MHz, DMSO-d_6): δ 171.3, 162.7, 162.3, 153.8, 152.7, 152.5, 142.3, 135.2, 128.7, 127.6, 123.3, 120.8, 114.7, 111.6, 111.3, 111.2, 103.8, 72.8, 55.8, 33.8; HREI MS:*m/z* calcd for $C_{20}H_{15}F_2N_5O_2S$ $[M]^+$ 427.43 Found 427.39.

3. 3-(4-(7H-imidazo[2,1-c][1,2,4]triazol-3-yl)-2-methoxyphenyl)-2-(2,4-difluorophenyl)thiazolidin-4-one

^{1}H NMR (600MHz, DMSO-d_6): δ 12.94 (s, 1H, NH-imidazole), 7.77 (d, J = 8.76 Hz, 1H, Ar-H), 7.44 (d, J = 7.38 Hz, 1H, Ar-H), 7.32 (s, 1H, Ar-H), 7.23(d, J = 6.87 Hz, 1H, Ar-H), 7.13 (d, J = 7.20 Hz, 1H, H-imidazole), 7.04 (d, J = 7.25 Hz, 1H, H-imidazole), 7.02 (d, J = 7.26 Hz, 1H, Ar-H), 7.00 (s, 1H, Ar-H), 6.49 (s, 1H, H-thiazolidinone), 3.95 (s, 2H, H-thiazolidinone), 3.85 (s, 3H, CH_3); ^{13}C NMR (150 MHz, DMSO-d_6): δ 171.2, 162.6, 159.4,

153.3, 152.6, 152.5, 135.2, 131.6, 128.5, 127.6, 123.3, 120.2, 114.2, 111.4, 111.2, 104.4, 100.1, 66.2, 55.3, 33.4; HREI MS:*m/z* calcd for $C_{20}H_{15}F_2N_5O_2S$ $[M]^+$ 427.43 Found 427.39.

4. 3-(4-(7H-imidazo[2,1-c][1,2,4]triazol-3-yl)-2-methoxyphenyl)-2-(4-hydroxyphenyl)thiazolidin-4-one

^{1}H NMR (600MHz, DMSO-*d6*): δ 12.91 (s, 1H, NH-imidazole), 9.21 (s, 1H, H-OH) 7.87 (d, *J* = 8.76 Hz, 1H, Ar-H), 7.64 (d, *J* = 7.98 Hz, 2H, Ar-H), 7.44 (d, *J* = 6.87 Hz, 1H, Ar-H), 7.31 (s, 1H, Ar-H), 7.13 (d, *J* = 6.75 Hz, 1H, H-imidazole), 7.05 (d, *J* = 7.26 Hz, 1H, H-imidazole), 7.02 (d, *J* = 7.25 Hz, 2H, Ar-H), 6.43 (s, 1H, H-thiazolidinone), 3.94 (s, 2H, H-thiazolidinone), 3.83 (s, 3H, CH_3); ^{13}C NMR (150 MHz, DMSO-d6): δ 171.4, 156.6, 153.2, 152.3, 152.1, 135.1, 131.4, 130.2, 130.1, 128.4, 127.2, 123.3, 120.1, 115.6, 115.4, 114.2, 111.3, 72.6, 55.5, 33.4; HREI MS:*m/z* calcd for $C_{20}H_{17}N_5O_3S$ $[M]^+$ 407.45 Found 407.41.

5. 3-(4-(7H-imidazo[2,1-c][1,2,4]triazol-3-yl)-2-methoxyphenyl)-2-(4-nitrophenyl)thiazolidin-4-one

^{1}H NMR (600MHz, DMSO-*d6*): δ 12.92 (s, 1H, NH-imidazole), 8.18 (d, *J* = 7.40 Hz, 2H, Ar-H), 7.75 (d, *J* = 8.46 Hz, 1H, Ar-H), 7.58 (d, *J* = 8.28 Hz, 2H, Ar-H), 7.43 (d, *J* = 7.20 Hz, 1H, Ar-H), 7.33 (s, 1H, Ar-H), 7.12 (d, *J* = 7.02 Hz, 1H, H-imidazole), 7.02 (d, *J* = 7.86 Hz, 1H, H-imidazole), 6.88 (s, 1H, H-thiazolidinone), 3.94 (s, 2H, H-thiazolidinone), 3.81 (s, 3H, CH_3); ^{13}C NMR (150 MHz, DMSO-d6): δ 171.1, 153.2, 152.4, 152.3, 144.2, 141.4, 135.1, 129.3, 129.2, 128.6, 127.2, 123.6, 123.3, 123.2, 120.2, 114.2, 111.4, 72.8, 55.8, 33.8; HREI MS:*m/z* calcd for $C_{20}H_{16}N_6O_4S$ $[M]^+$ 436.45 Found 436.41.

6. 3-(4-(7H-imidazo[2,1-c][1,2,4]triazol-3-yl)-2-methoxyphenyl)-2-(4-chlorophenyl)thiazolidin-4-one

^{1}H NMR (600MHz, DMSO-*d6*): δ 12.74 (s, 1H, NH-imidazole), 7.75 (d, *J* = 6.75 Hz, 1H, Ar-H), 7.58 (d, *J* = 7.56 Hz, 1H, Ar-H), 7.43 (d, *J* = 7.57 Hz, 2H, Ar-H), 7.33 (s, 1H, Ar-H), 7.20 (d, *J* = 7.38 Hz, 2H, Ar-H), 7.14 (d, *J* = 7.08 Hz, 1H, H-imidazole), 7.03 (d, *J* = 7.26 Hz, 1H, H-imidazole), 6.44 (s, 1H, H-thiazolidinone), 3.93 (s, 2H, H-thiazolidinone), 3.84 (s, 3H, CH_3); ^{13}C NMR (150 MHz, DMSO-d6): δ 171.4, 153.2, 152.4, 152.3, 137.2, 135.1, 131.5,

129.3, 129.0, 128.7, 127.6, 127.4, 123.5, 123.2, 120.2, 114.5, 111.5, 72.3, 55.4, 33.5; HREI MS:*m/z* calcd for $C_{20}H_{16}ClN_5O_2S$ $[M]^+$ 425.89 Found 425.85.

7. 3-(4-(7H-imidazo[2,1-c][1,2,4]triazol-3-yl)-2-methoxyphenyl)-2-(3,4-dichlorophenyl)thiazolidin-4-one

^{1}H NMR (600MHz, DMSO-d_6): δ 12.90 (s, 1H, NH-imidazole), 7.75 (d, *J* = 8.94 Hz, 1H, Ar-H), 7.63 (d, *J* = 8.16 Hz, 1H, Ar-H), 7.42 (d, *J* = 8.16 Hz, 1H, Ar-H), 7.38 (s, 1H, Ar-H), 7.31 (s, 1H, Ar-H), 7.12 (d, *J* = 8.58 Hz, 1H, H-imidazole), 7.09 (d, *J* = 7.86 Hz, 1H, Ar-H), 7.06 (d, *J* = 7.86 Hz, 1H, H-imidazole), 6.45 (s, 1H, H-thiazolidinone), 3.96 (s, 2H, H-thiazolidinone), 3.84 (s, 3H, CH_3); ^{13}C NMR (150 MHz, DMSO-d_6): δ 171.2, 153.4, 152.5, 152.2, 138.6, 135.4, 131.6, 131.4, 130.2, 129.5, 128.4, 128.3, 127.2, 123.2, 120.4, 114.2, 111.3, 72.6, 55.1, 33.2; HREI MS:*m/z* calcd for $C_{20}H_{15}Cl_2N_5O_2S$ $[M]^+$ 460.33 Found 460.29.

8. 3-(4-(7H-imidazo[2,1-c][1,2,4]triazol-3-yl)-2-methoxyphenyl)-2-(4-chloro-2-nitrophenyl)thiazolidin-4-one

^{1}H NMR (600MHz, DMSO-d_6): δ 12.93 (s, 1H, NH-imidazole), 8.24 (s, 1H, Ar-H), 7.77 (d, *J* = 8.46 Hz, 1H, Ar-H), 7.74 (d, *J* = 7.75 Hz, 1H, Ar-H), 7.46 (d, *J* = 6.80 Hz, 2H, Ar-H), 7.33 (s, 1H, Ar-H), 7.14 (d, *J* = 6.50 Hz, 1H, H-imidazole), 7.05 (d, *J* = 7.28 Hz, 1H, H-imidazole), 6.41 (s, 1H, H-thiazolidinone), 3.94 (s, 2H, H-thiazolidinone), 3.87 (s, 3H, CH_3); ^{13}C NMR (150 MHz, DMSO-d_6): δ 171.2, 153.4, 152.5, 152.4, 150.2, 135.1, 134.5, 132.1, 130.2, 130.1, 128.6, 127.3, 124.2, 123.5, 120.4, 114.3, 111.2, 68.2, 55.4, 33.5; HREI MS:*m/z* calcd for $C_{20}H_{15}ClN_6O_4S$ $[M]^+$ 470.89 Found 470.85.

9. 3-(4-(7H-imidazo[2,1-c][1,2,4]triazol-3-yl)-2-methoxyphenyl)-2-(3-fluoro-5-hydroxyphenyl)thiazolidin-4-one

^{1}H NMR (600MHz, DMSO-d_6): δ 12.92 (s, 1H, NH-imidazole), 9.42 (s, 1H, H-OH), 7.74 (d, *J* = 7.56 Hz, 1H, Ar-H), 7.41 (d, *J* = 7.50 Hz, 1H, Ar-H), 7.39 (s, 1H, Ar-H), 7.12 (d, *J* = 6.54 Hz, 1H, H-imidazole), 7.04 (d, *J* = 7.74 Hz, 1H, H-imidazole), 7.02 (s, 1H, Ar-H), 6.88 (s, 1H, Ar-H), 6.73 (s, 1H, Ar-H), 6.44 (s, 2H, H-thiazolidinone), 3.97 (s, 2H, H-thiazolidinone),

3.86 (s, 3H, CH_3); ^{13}C NMR (150 MHz, DMSO-d_6): δ 171.3, 164.7, 160.7, 153.8, 152.7, 152.5, 142.3, 135.8, 128.4, 127.5, 123.3, 120.5, 114.7, 111.6, 110.3, 108.3, 100.8, 73.1, 55.7, 33.8; HREI MS:*m/z* calcd for $C_{20}H_{16}FN_5O_3S$ $[M]^+$ 425.44 Found 425.40.

10. 3-(4-(7H-imidazo[2,1-c][1,2,4]triazol-3-yl)-2-methoxyphenyl)-2-(4-(trifluoromethyl)phenyl)thiazolidin-4-one

^1H NMR (600MHz, DMSO-d_6): δ 12.87 (s, 1H, NH-imidazole), 7.79 (d, J = 8.34 Hz, 1H, Ar-H), 7.56 (d, J = 7.26 Hz, 2H, Ar-H), 7.42 (d, J = 6.91 Hz, 1H, Ar-H), 7.32 (s, 1H, Ar-H), 7.22 (d, J = 8.70 Hz, 2H, Ar-H), 7.14 (d, J = 8.99 Hz, 1H, H-imidazole), 7.03 (d, J = 7.20 Hz, 1H, H-imidazole), 6.42 (s, 1H, H-thiazolidinone), 3.94 (s, 2H, H-thiazolidinone), 3.81 (s, 3H, CH_3); ^{13}C NMR (150 MHz, DMSO-d_6): δ 171.2, 153.4, 152.5, 152.4, 142.5, 135.1, 129.5, 129.3, 129.2, 128.7, 128.6, 127.3, 124.3, 124.2, 123.5, 120.3, 114.3, 111.2, 72.6, 55.7, 33.3; HREI MS:*m/z* calcd for $C_{21}H_{16}F_3N_5O_2S$ $[M]^+$ 459.45 Found 459.41.

11. 3-(4-(7H-imidazo[2,1-c][1,2,4]triazol-3-yl)-2-methoxyphenyl)-2-(4-fluoro-2-hydroxyphenyl)thiazolidin-4-one

^1H NMR (600MHz, DMSO-d_6): δ 12.95 (s, 1H, NH-imidazole), 9.75 (s, 1H, H-OH), 7.74 (d, J = 7.02 Hz, 1H, Ar-H), 7.44 (d, J = 7.26 Hz, 1H, Ar-H), 7.33 (s, 1H, Ar-H), 7.12 (d, J = 7.50 Hz, 1H, H-imidazole), 7.08 (d, J = 8.99 HZ, 1H, Ar-H), 7.04 (d, J = 8.58 Hz, 1H, H-imidazole), 7.01 (s, 1H, Ar-H), 6.85 (d, J = 7.26 Hz, 1H, Ar-H), 6.43 (s, 1H, H-thiazolidinone), 3.96 (s, 2H, H-thiazolidinone), 3.87 (s, 3H, CH_3); ^{13}C NMR (150 MHz, DMSO-d_6): δ 171.7, 162.6, 156.2, 153.2, 152.4, 152.2, 135.3, 131.2, 128.5, 127.2, 123.3, 120.4, 120.1, 115.5, 115.4, 111.9, 104.2, 72.8, 55.8, 33.8; HREI MS:*m/z* calcd for $C_{20}H_{16}FN_5O_3S$ $[M]^+$ 425.44 Found 425.40.

12. 3-(4-(7H-imidazo[2,1-c][1,2,4]triazol-3-yl)-2-methoxyphenyl)-2-(4-fluoro-3-hydroxyphenyl)thiazolidin-4-one

^1H NMR (600MHz, DMSO-d_6): δ 12.93 (s, 1H, NH-imidazole), 9.03 (s, 1H, H-OH), 7.76 (d, J = 7.08 Hz, 1H, Ar-H), 7.45 (d, J = 6.75 Hz, 1H, Ar-H), 7.37 (s, 1H, Ar-H), 7.13 (d, J = 7.14 Hz, 1H, H-imidazole), 7.05 (d, J = 7.98 Hz, 1H, Ar-H), 7.03 (d, J = 8.10 Hz, 1H, H-imidazole), 6.93 (d, J = 7.08 Hz, 1H, Ar-H), 6.88 (s, 1H, Ar-H), 6.42 (s, 1H, H-

thiazolidinone), 3.95 (s, 2H, H-thiazolidinone), 3.85 (s, 3H, CH_3); ^{13}C NMR (150 MHz, DMSO-d_6): δ 171.2, 153.4, 152.2, 152.1, 152.0, 143.6, 135.7, 131.6, 128.3, 127.2, 123.3, 120.7, 120.6, 115.5, 115.2, 114.8, 104.3, 72.6, 53.3, 33.7; HREI MS:*m/z* calcd for $C_{20}H_{16}FN_5O_3S$ $[M]^+$ 425.44 Found 425.40.

13. 3-(4-(7H-imidazo[2,1-c][1,2,4]triazol-3-yl)-2-methoxyphenyl)-2-(4-fluoro-3-nitrophenyl)thiazolidin-4-one

^1H NMR (600MHz, DMSO-d_6): δ 12.99 (s, 1H, NH-imidazole), 8.18 (s, 1H, Ar-H), 7.73 (d, J = 7.08 Hz, 1H, Ar-H), 7.63 (d, J = 8.28 Hz, 1H, Ar-H), 7.47 (d, J = 6.78 Hz, 2H, Ar-H), 7.34 (s, 1H, Ar-H), 7.17 (d, J = 7.02 Hz, 1H, H-imidazole), 7.08 (d, J = 7.14 Hz, 1H, H-imidazole), 6.41 (s, 1H, H-thiazolidinone), 3.96 (s, 2H, H-thiazolidinone), 3.83 (s, 3H, CH_3); ^{13}C NMR (150 MHz, DMSO-d_6): δ 171.5, 153.6, 153.2, 152.4, 152.3, 136.5, 135.1, 131.3, 129.6, 128.7, 127.6, 126.4, 123.5, 123.2, 120.2, 114.5, 111.5, 71.3, 55.4, 33.5; HREI MS:*m/z* calcd for $C_{20}H_{15}FN_6O_4S$ $[M]^+$ 454.44 Found 454.40.

14. 3-(4-(7H-imidazo[2,1-c][1,2,4]triazol-3-yl)-2-methoxyphenyl)-2-(4-methoxyphenyl)thiazolidin-4-one

^1H NMR (600MHz, DMSO-d_6): δ 12.94 (s, 1H, NH-imidazole), 7.80 (d, J = 7.68 Hz, 2H, Ar-H), 7.63 (d, J = 8.28 Hz, 1H, Ar-H), 7.47 (d, J = 8.99 Hz, 1H, Ar-H), 7.32 (s, 1H, Ar-H), 7.14 (d, J = 8.22 Hz, 1H, H-imidazole), 7.08 (d, J = 7.25 Hz, 1H, H-imidazole), 6.90 (d, J = 6.69 Hz, 2H, Ar-H), 6.43 (s, 1H, H-thiazolidinone), 3.95 (s, 2H, H-thiazolidinone), 3.85 (s, 3H, CH_3), 3.80 (s, 3H, CH_3); ^{13}C NMR (150 MHz, DMSO-d_6): δ 171.4, 160.7, 153.8, 152.7, 152.5, 135.8, 128.4, 127.5, 123.3, 120.5, 127.3, 114.7, 111.6, 114.3, 110.3, 108.3, 100.8, 71.1, 55.7, 55.5, 33.3; HREI MS:*m/z* calcd for $C_{21}H_{19}N_5O_3S$ $[M]^+$ 421.48 Found 421.44.

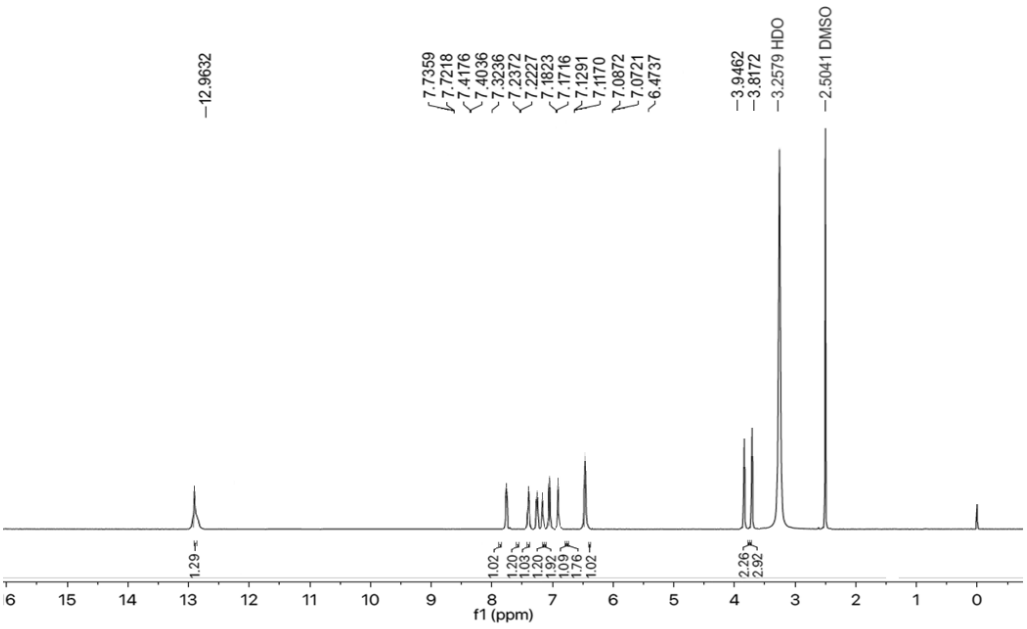

Figure-S.1 Proton spectral analysis of compound-1

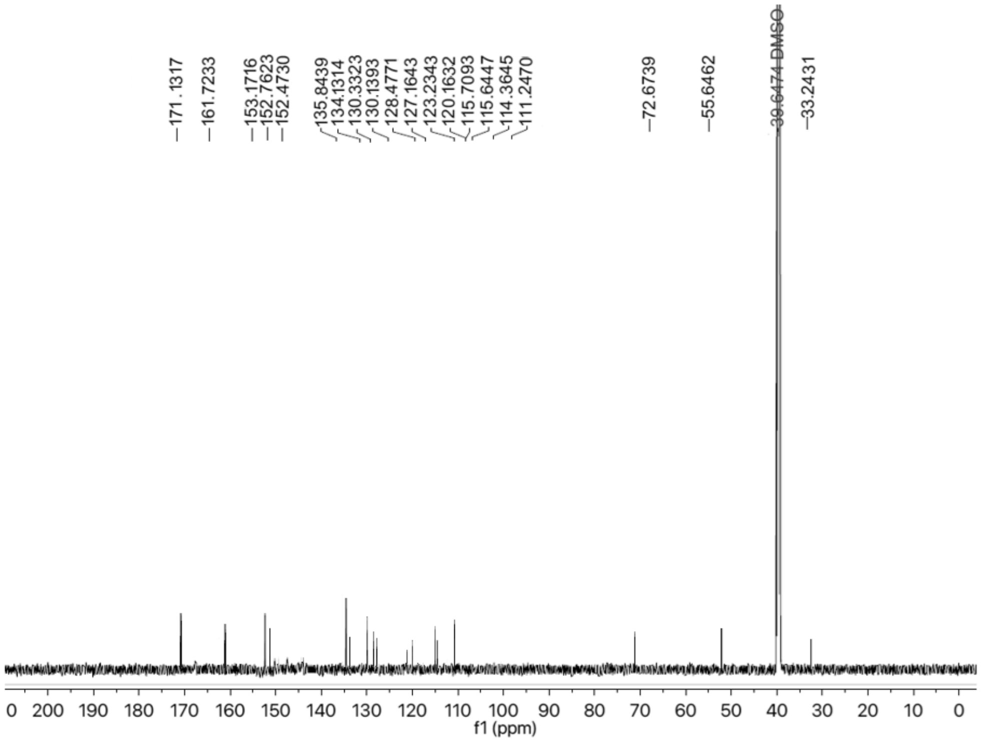

Figure-S.2 Carbon spectral analysis of compound-1

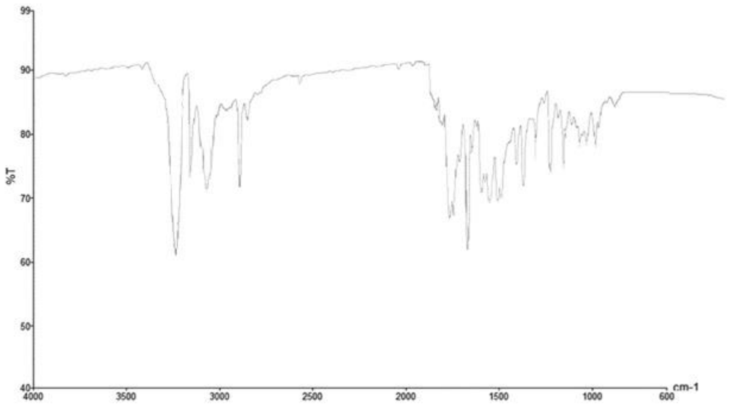

Figure-S.3 IR analysis of compound-1

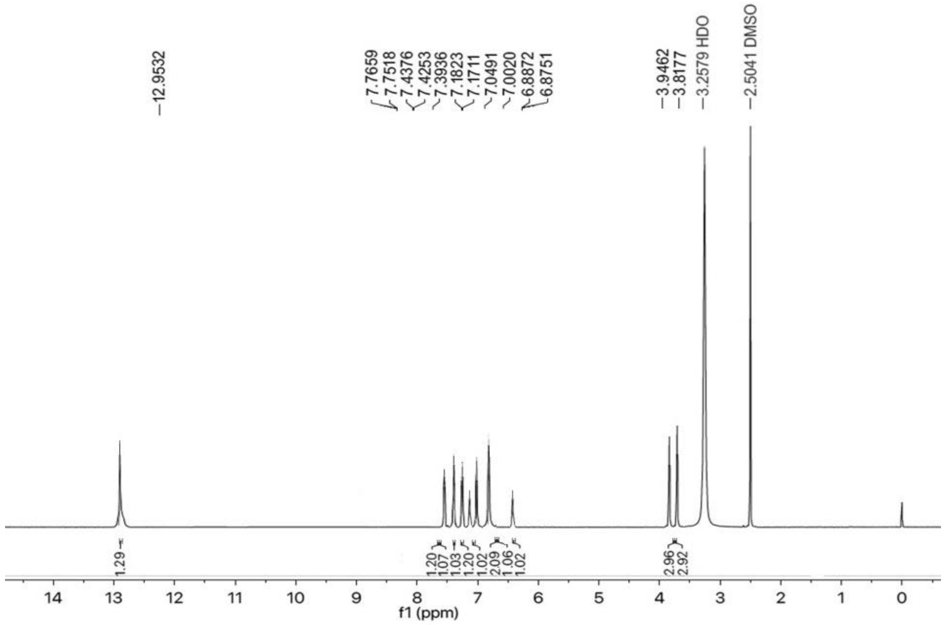

Figure-S.4 Proton spectral analysis of compound-2

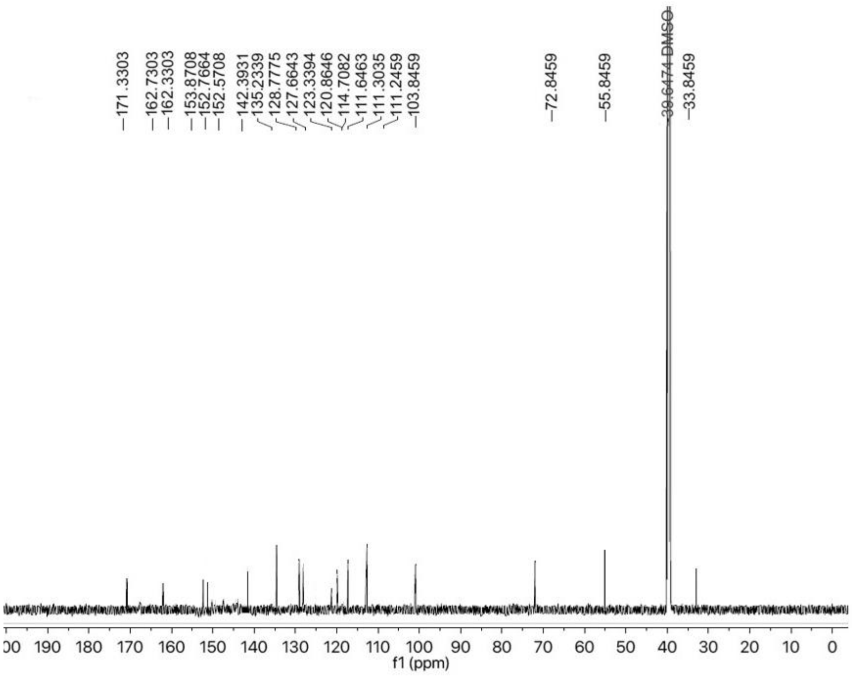

Figure-S.5 Carbon spectral analysis of compound-2

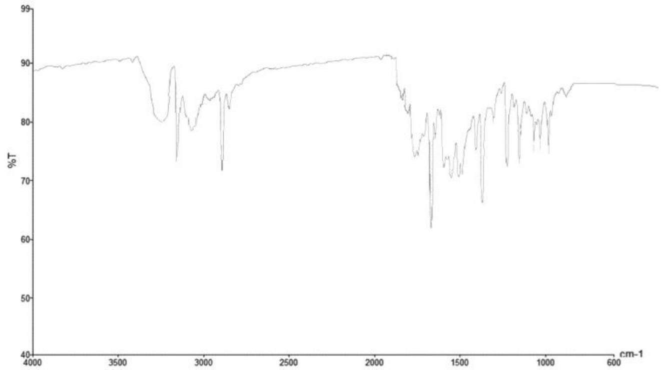

Figure-S.6 IR analysis of compound-2

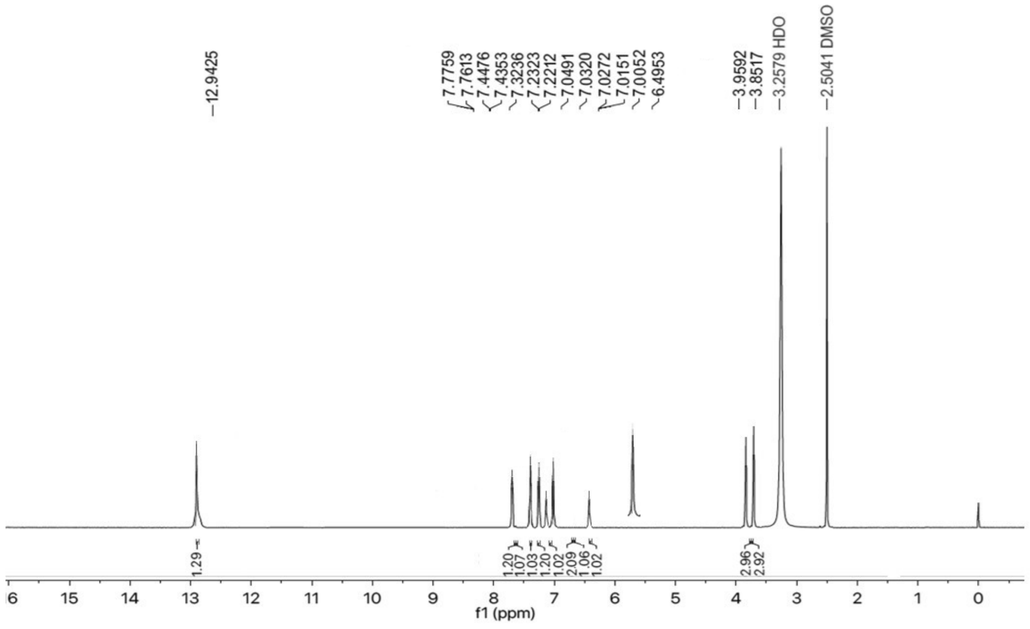

Figure-S.7 Proton spectral analysis of compound-3

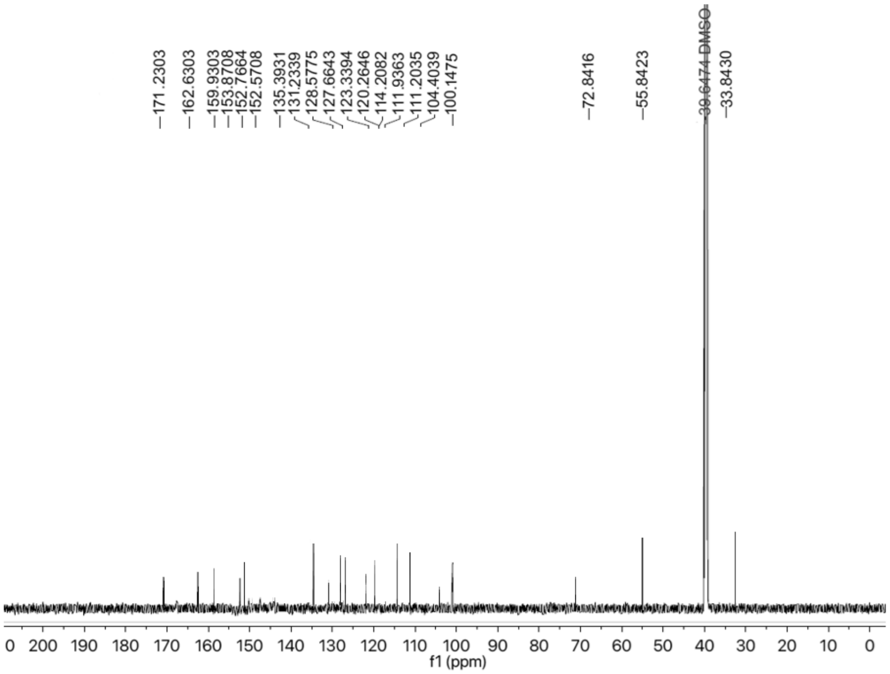

Figure-S.8 Carbon spectral analysis of compound-3

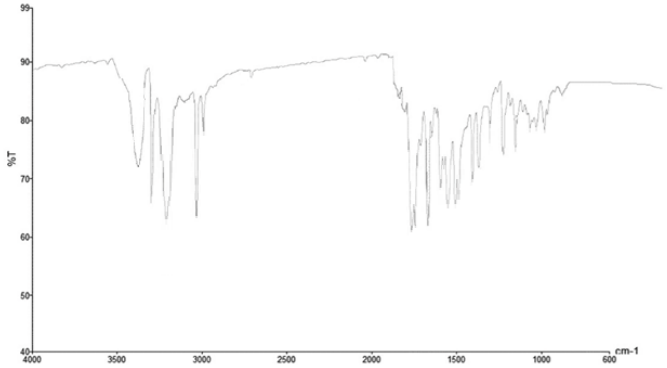

Figure-S.9 IR analysis of compound-3

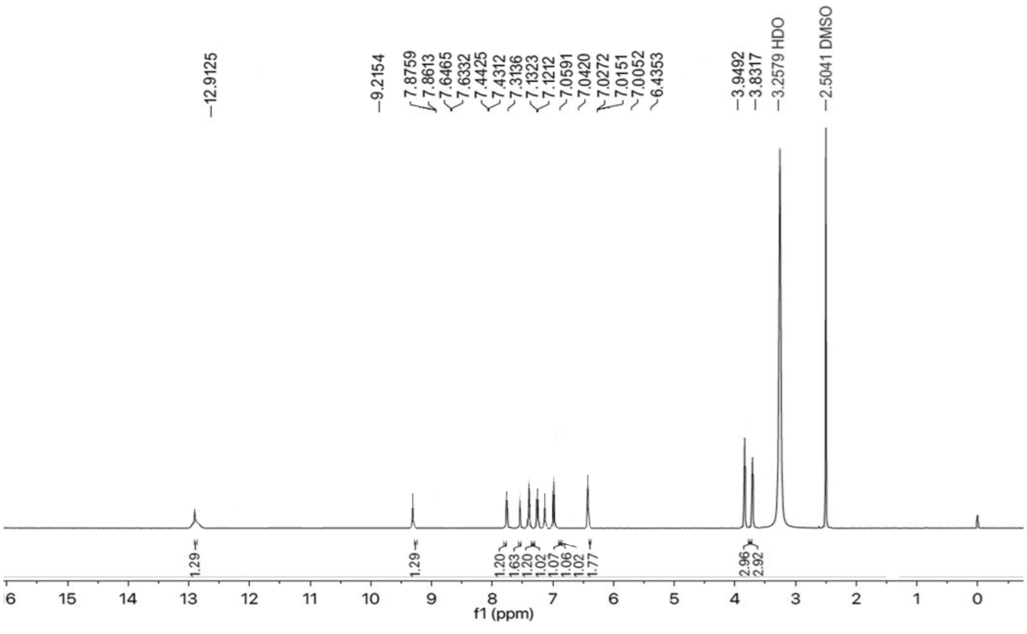

Figure-S.10 Proton spectral analysis of compound-4

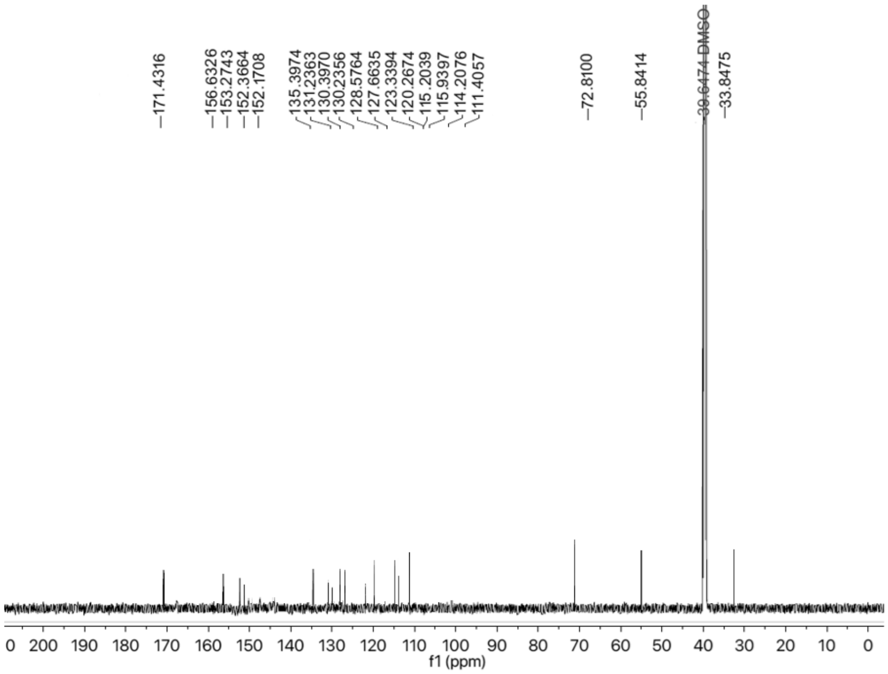

Figure-S.11 Carbon spectral analysis of compound-4

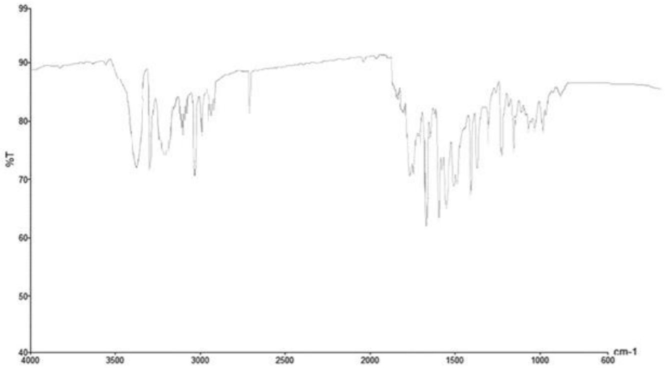

Figure-S.12 IR analysis of compound-4

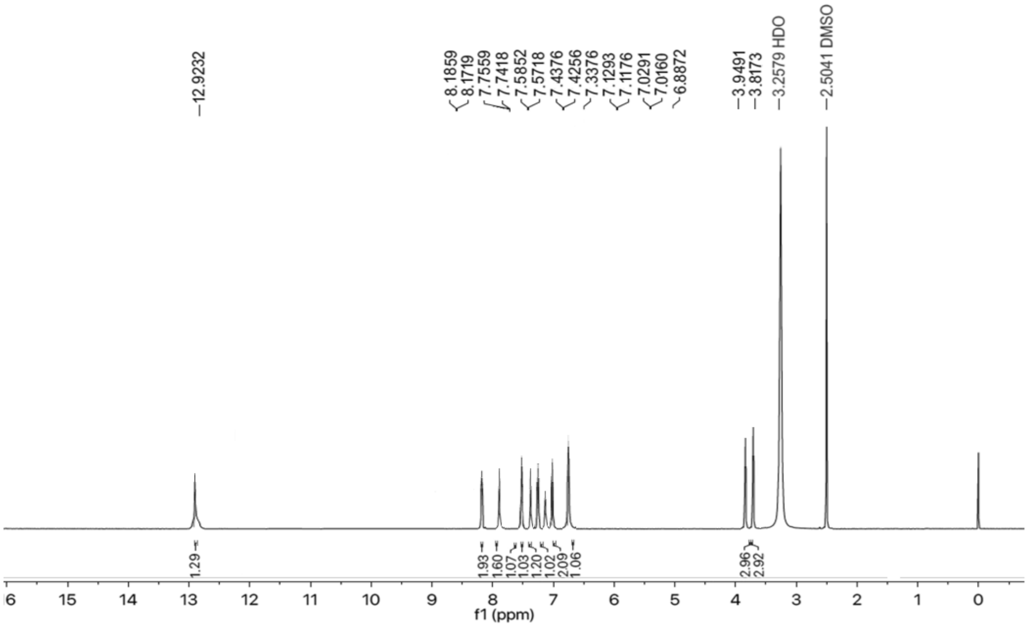

Figure-S.13 Proton spectral analysis of compound-5

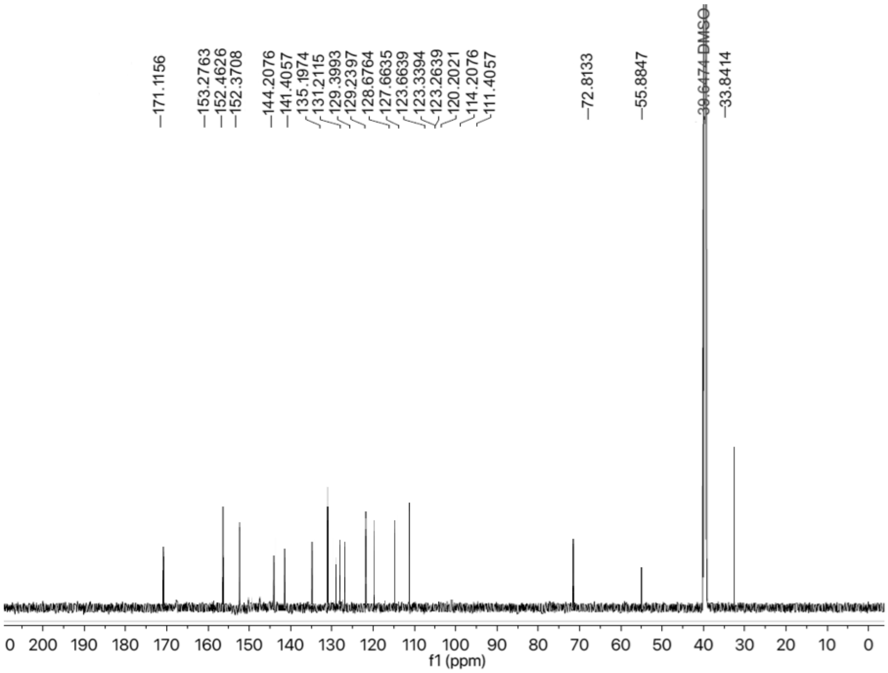

Figure-S.14 Carbon spectral analysis of compound-5

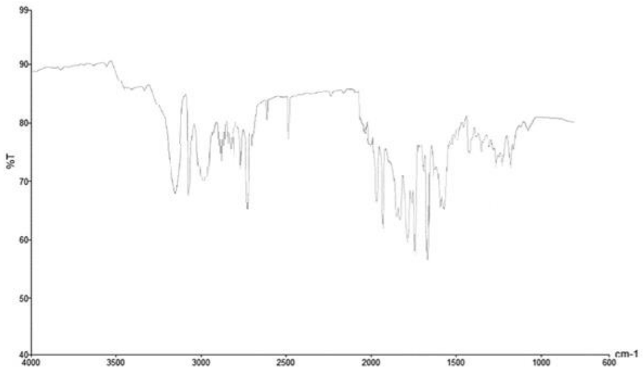

Figure-S.15 IR analysis of compound-5

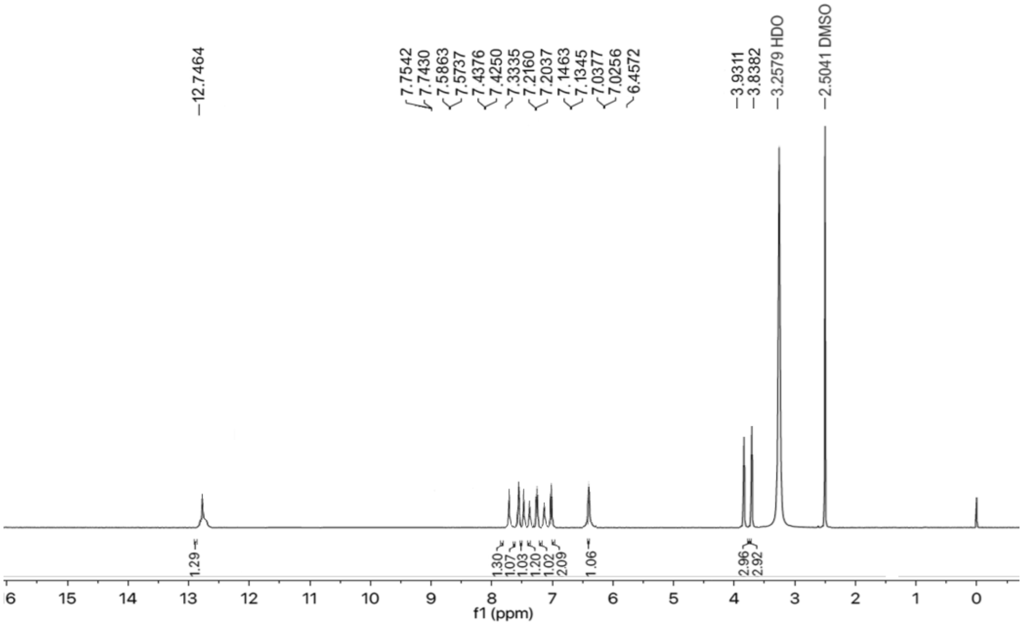

Figure-S.16 Proton spectral analysis of compound-6

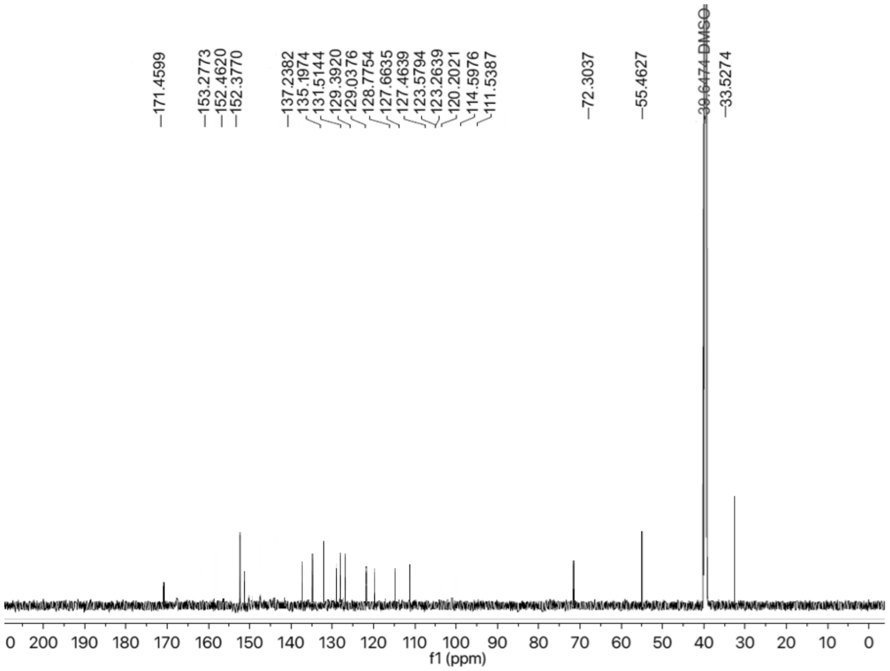

Figure-S.17 Carbon spectral analysis of compound-6

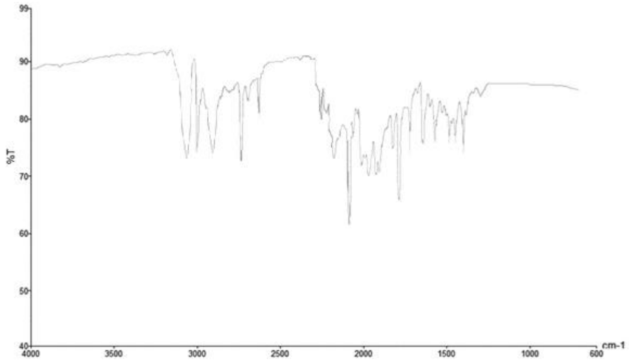

Figure-S.18 IR analysis of compound-6

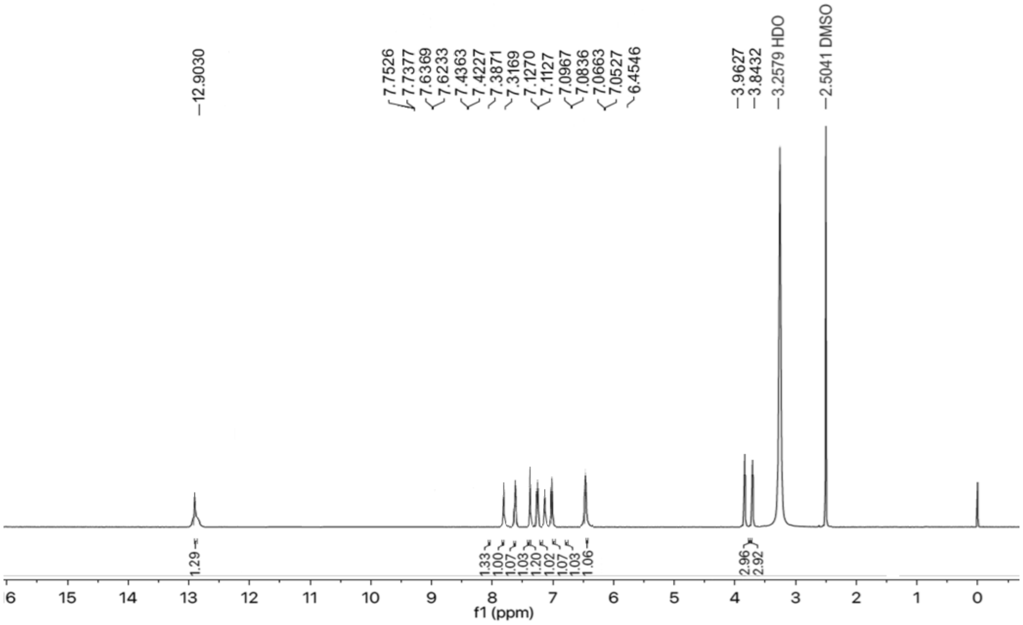

Figure-S.19 Proton spectral analysis of compound-7

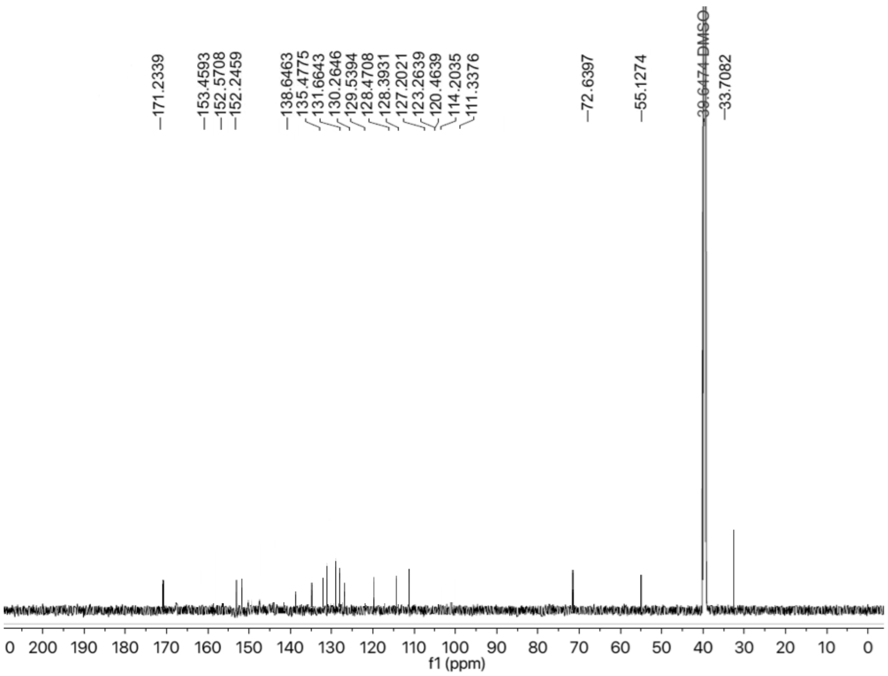

Figure-S.20 Carbon spectral analysis of compound-7

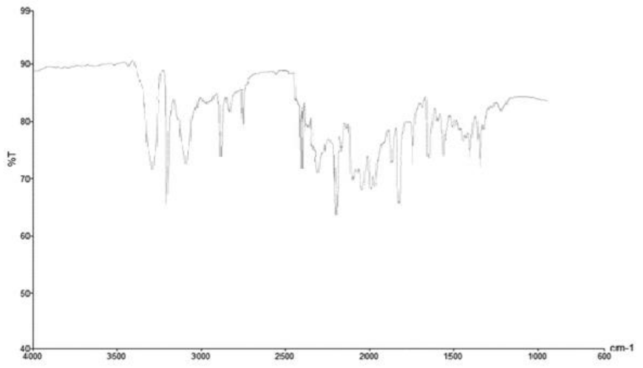

Figure-S.21 IR analysis of compound-7

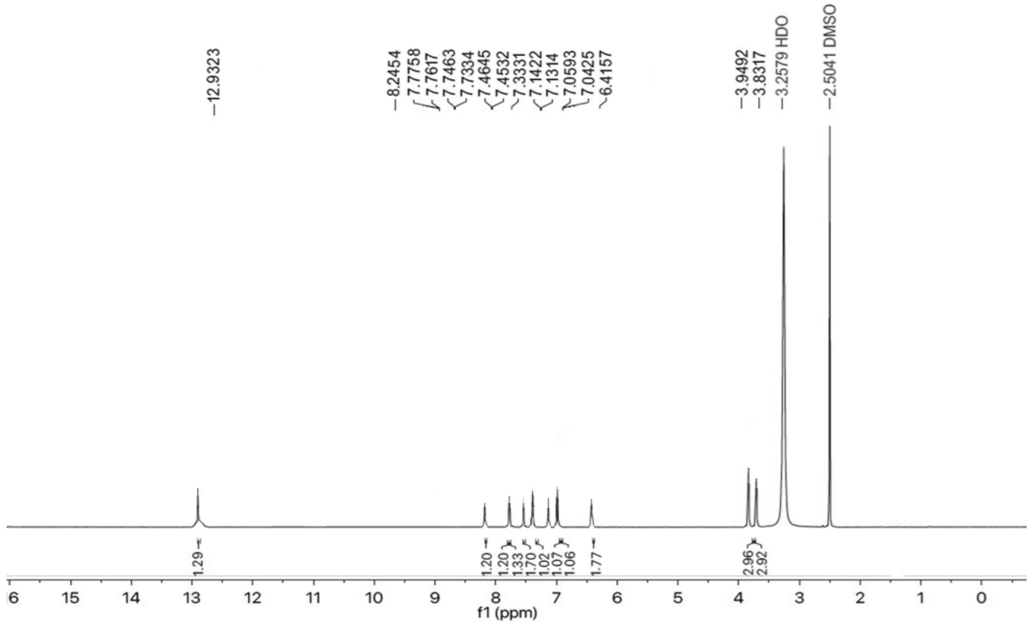

Figure-S.22 Proton spectral analysis of compound-8

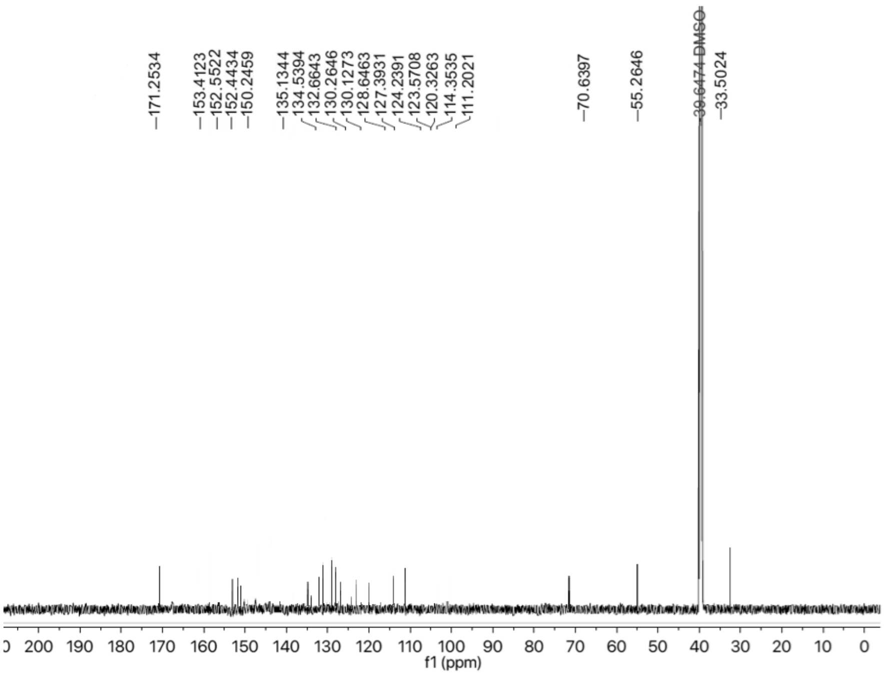

Figure-S.23 Carbon spectral analysis of compound-8

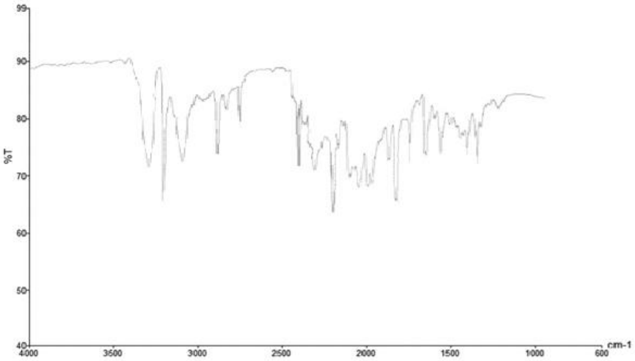

Figure-S.24 IR analysis of compound-8

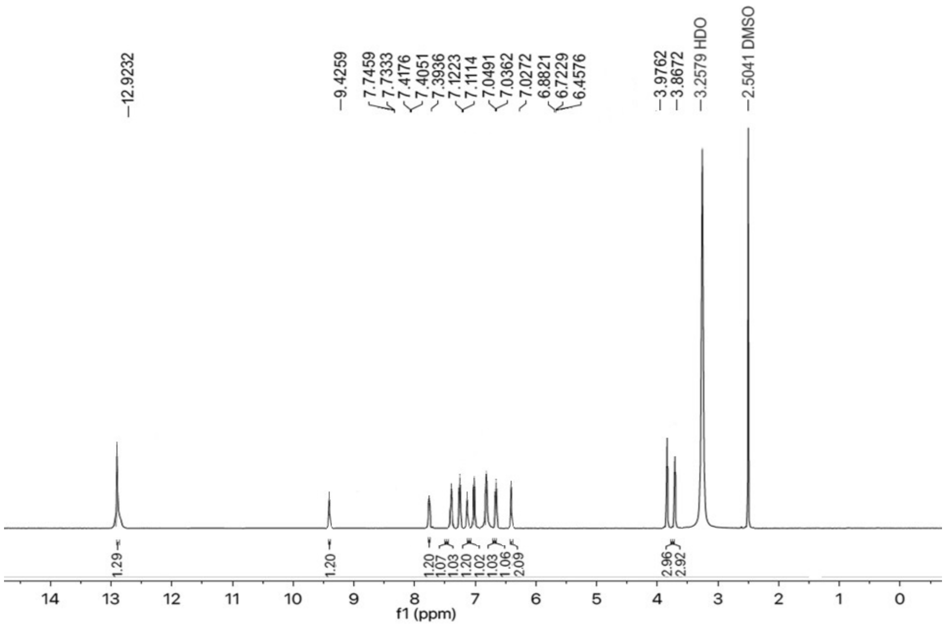

Figure-S.25 Proton spectral analysis of compound-9

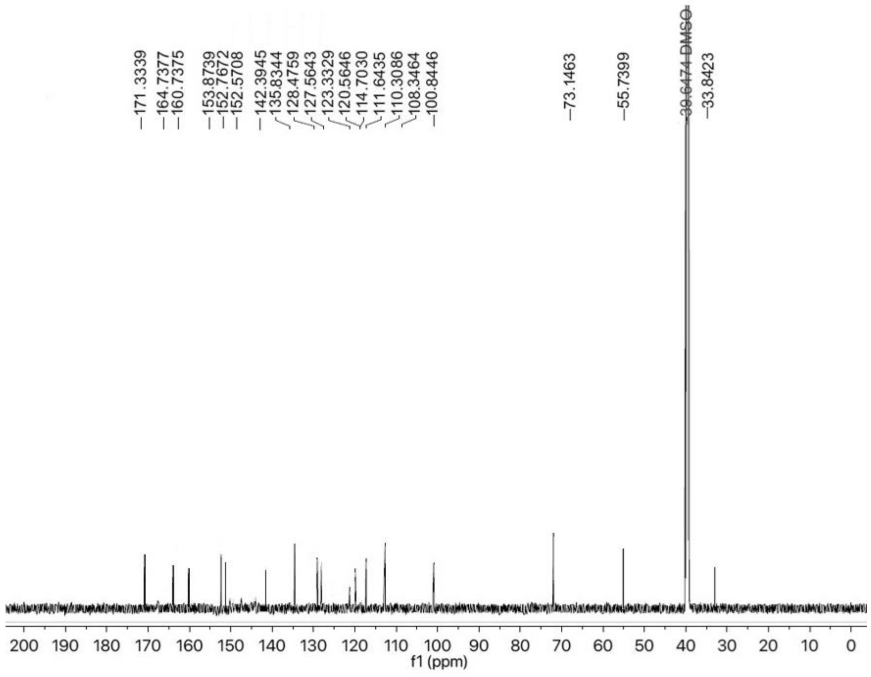

Figure-S.26 Carbon spectral analysis of compound-9

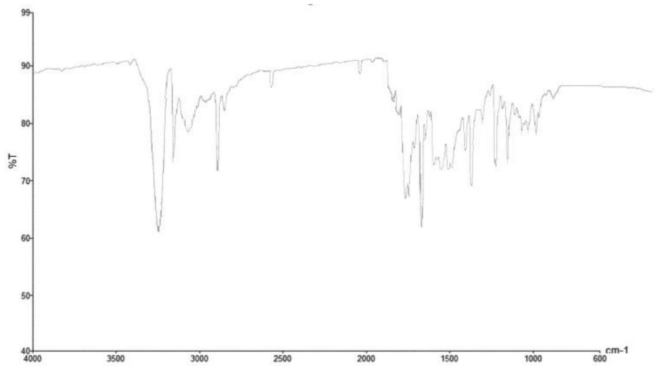

Figure-S.27 IR analysis of compound-9

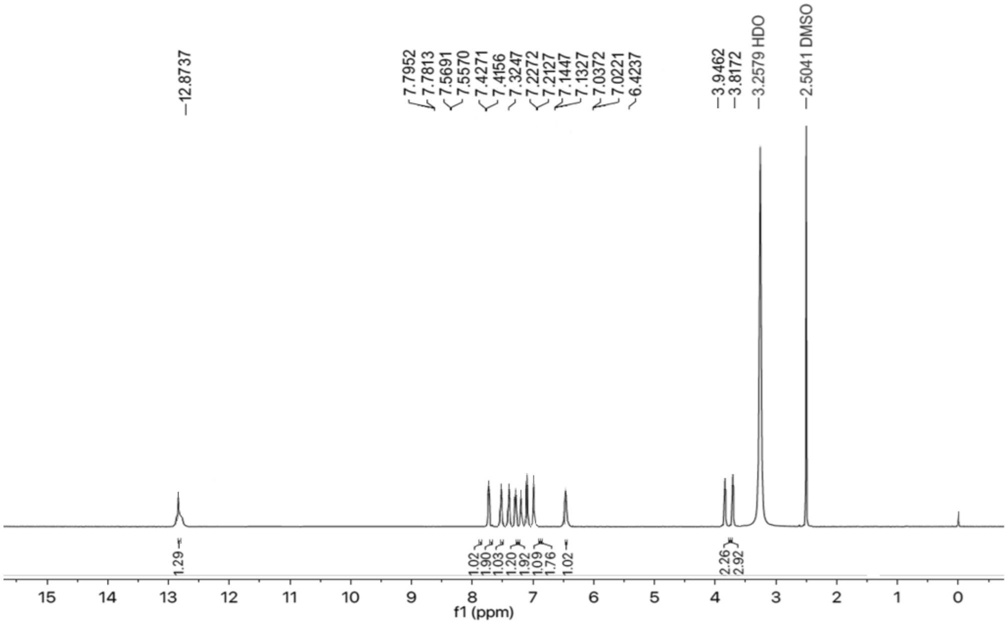

Figure-S.28 Proton spectral analysis of compound-10

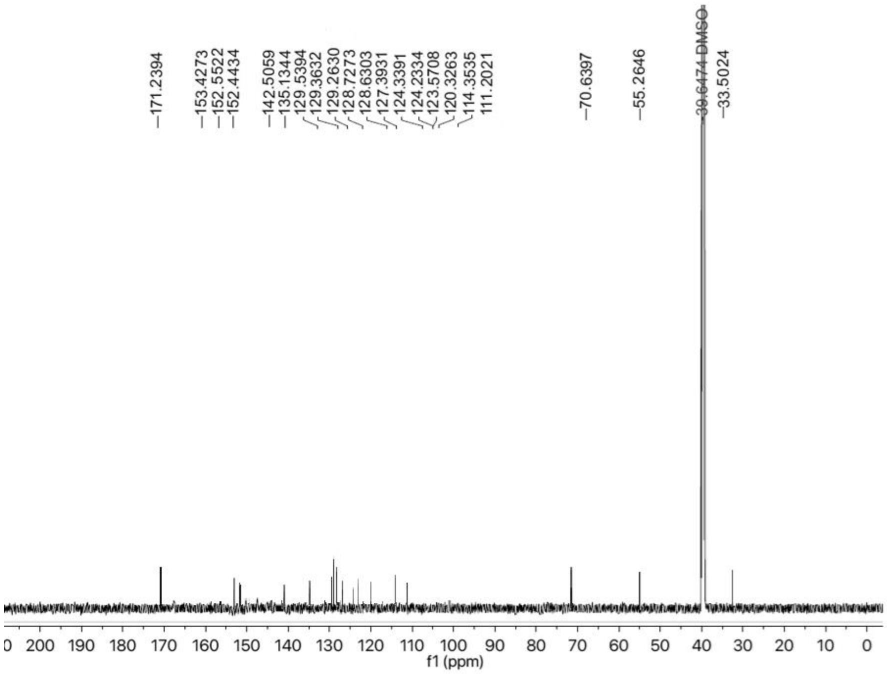

Figure-S.29 Carbon spectral analysis of compound-10

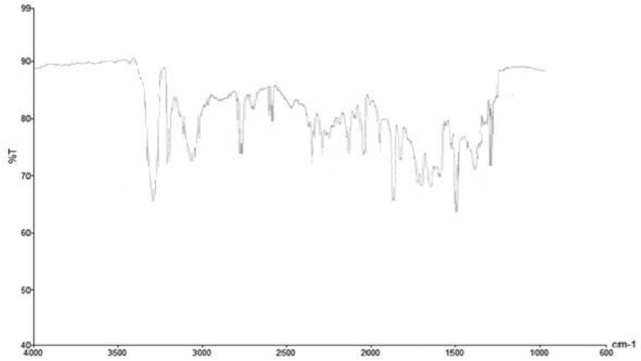

Figure-S.30 IR analysis of compound-10

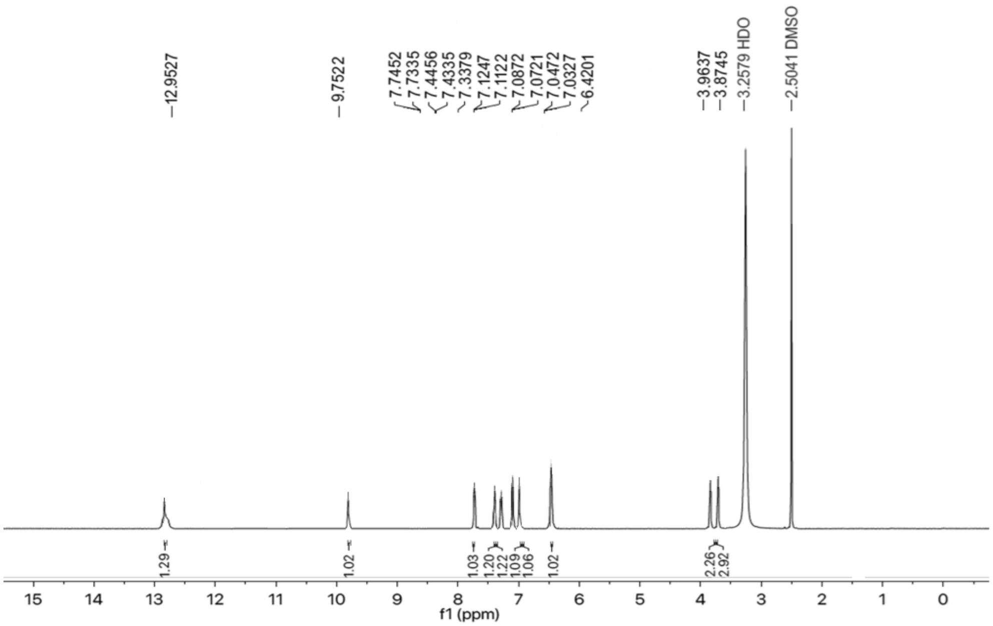

Figure-S.31 Proton spectral analysis of compound-11

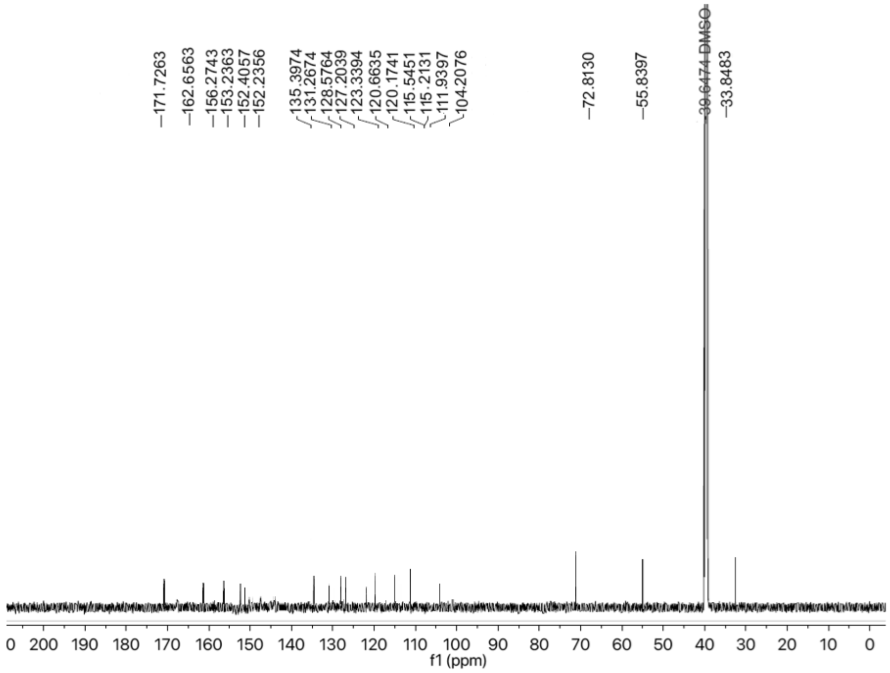

Figure-S.32 Carbon spectral analysis of compound-11

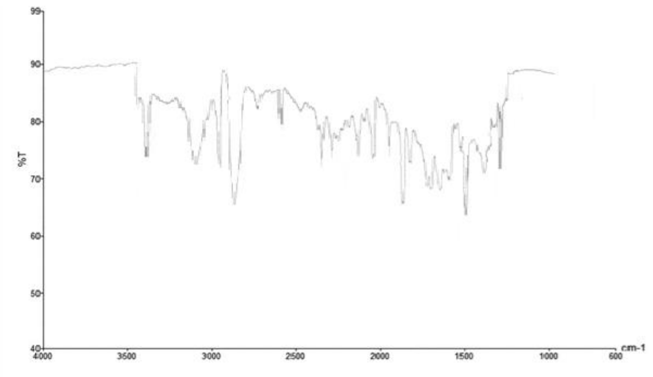

Figure-S.33 IR analysis of compound-11

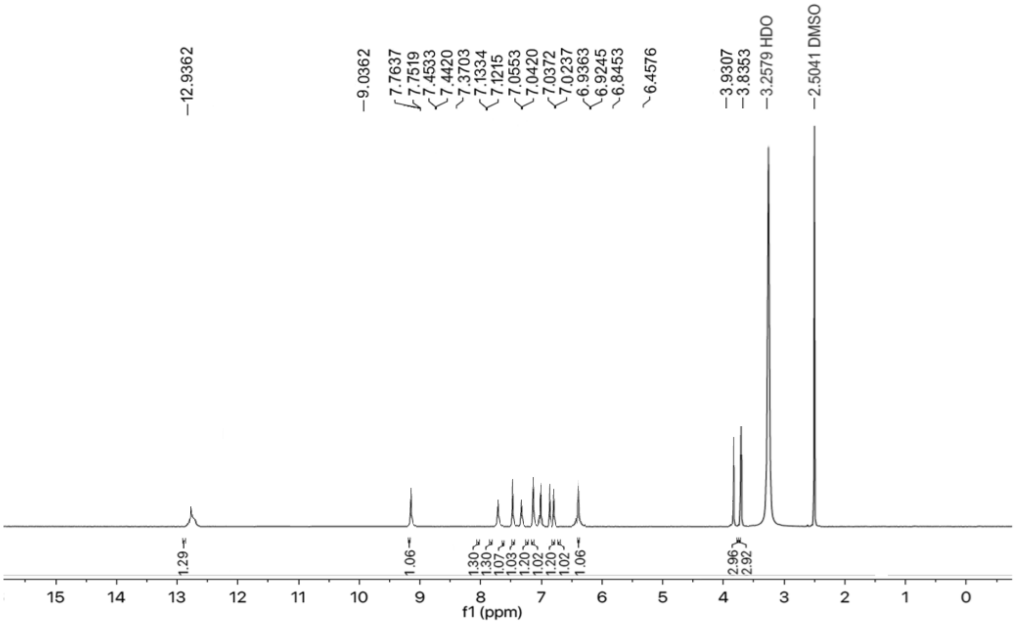

Figure-S.34 Proton spectral analysis of compound-12

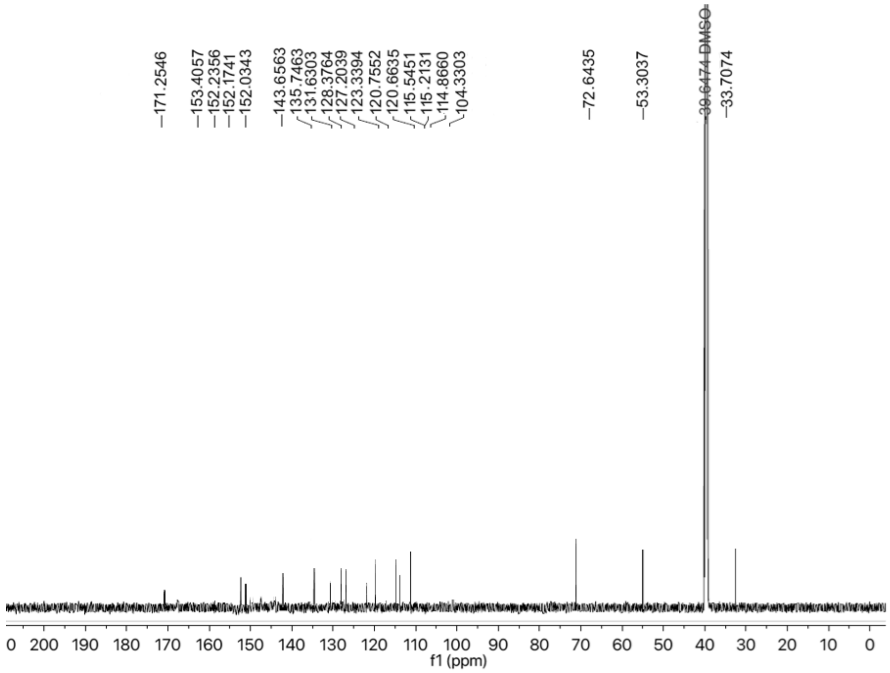

Figure-S.35 Carbon spectral analysis of compound-12

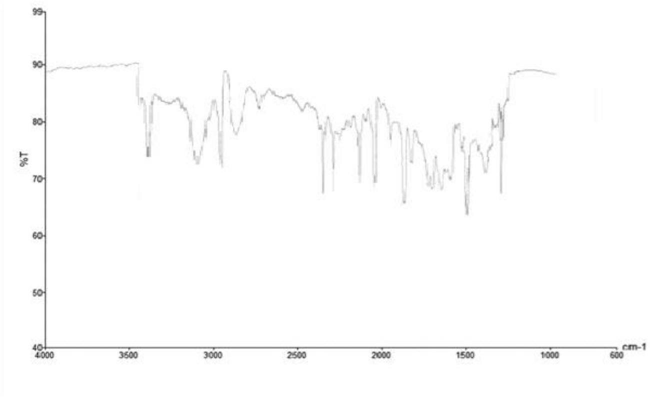

Figure-S.36 IR analysis of compound-12

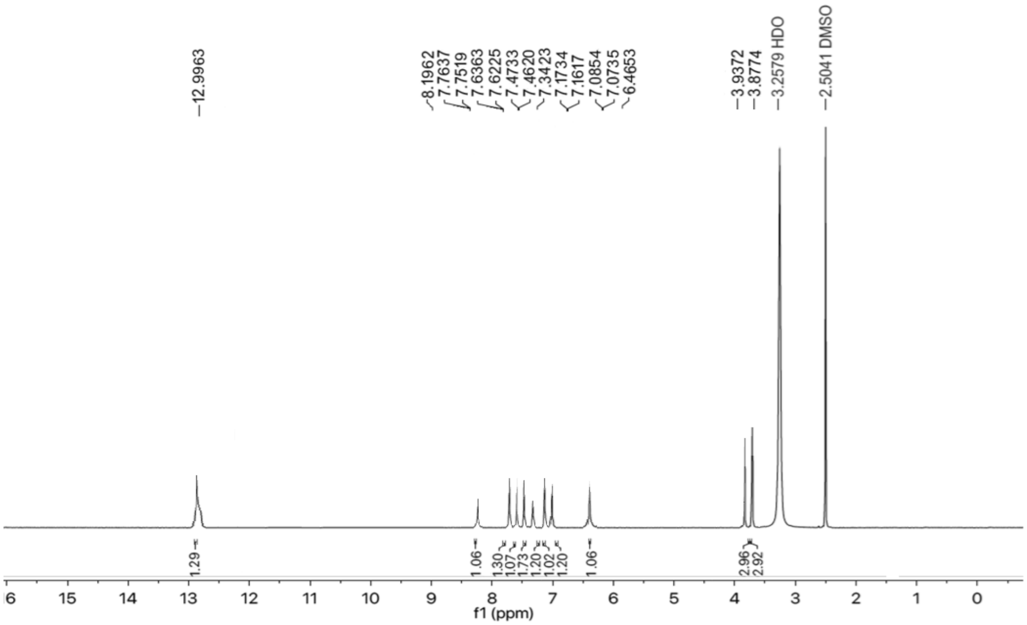

Figure-S.37 Proton spectral analysis of compound-13

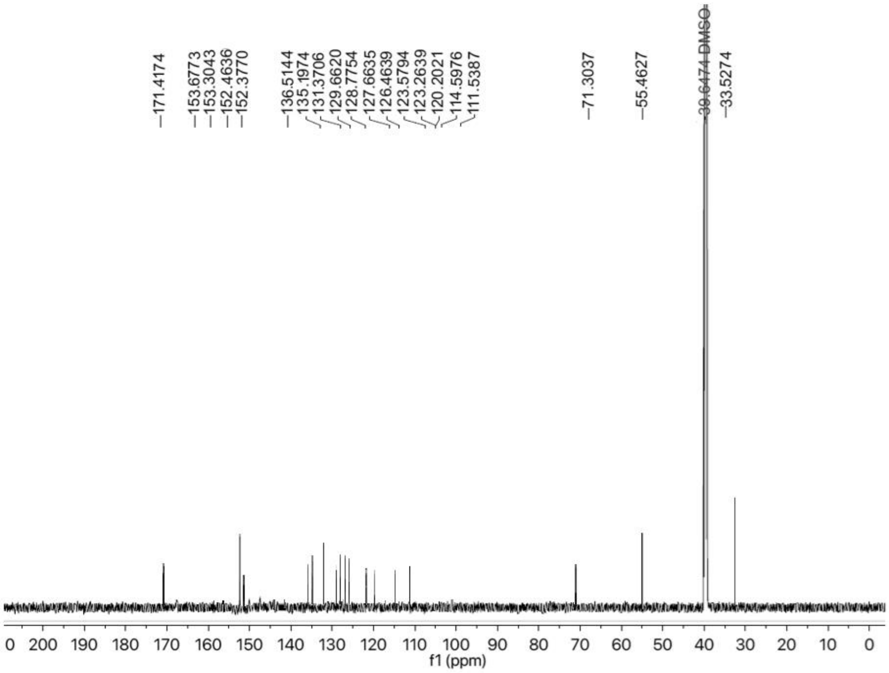

Figure-S.38 Carbon spectral analysis of compound-13

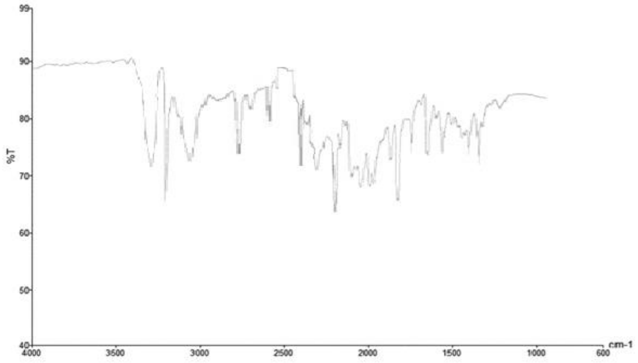

Figure-S.39 IR analysis of compound-13

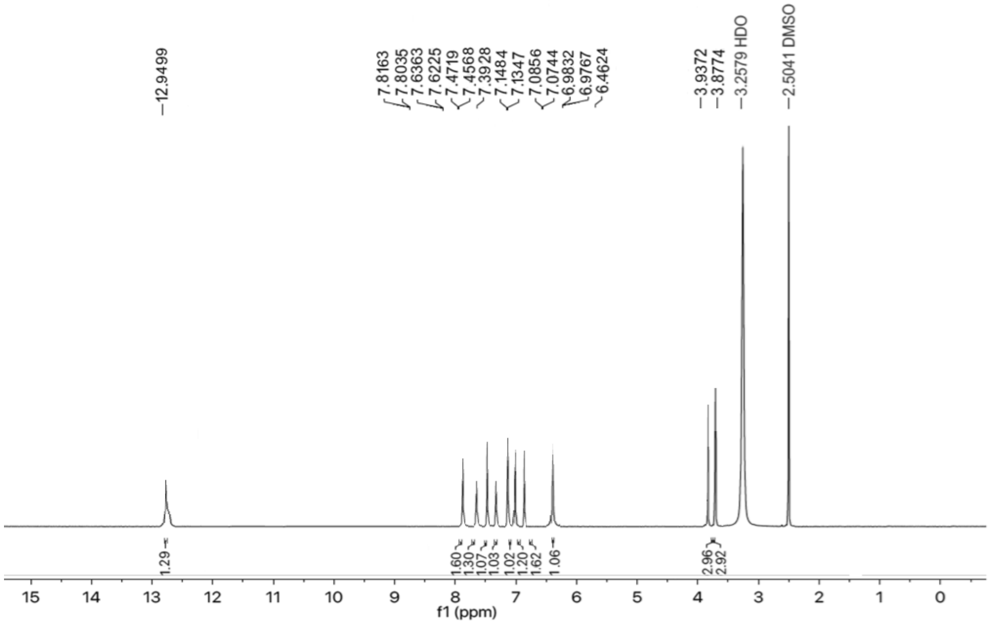

Figure-S.40 Proton spectral analysis of compound-14

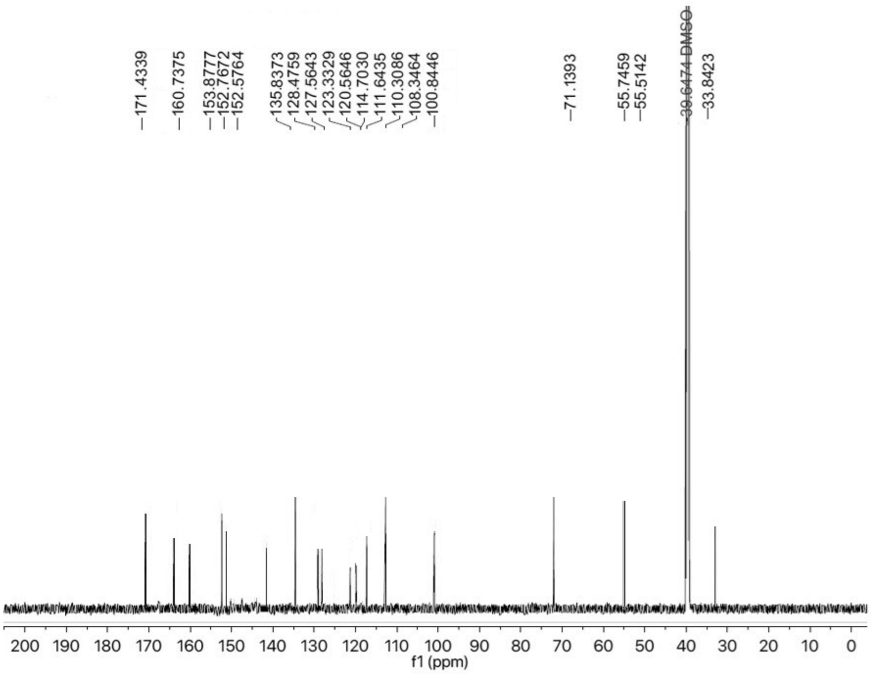

Figure-S.41 Carbon spectral analysis of compound-14

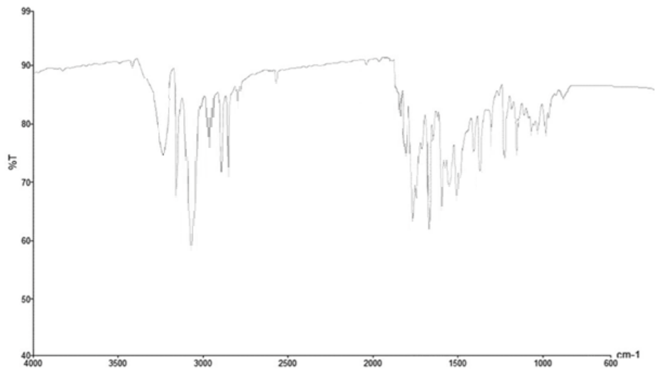

Figure-S.42 IR analysis of compound-14

S.2 AChE and BuChE assay protocols

With a little bit modification, inhibitory potential against AChE and BuChE was investigated according to the reported protocol [44]. The total volume of the reaction mixture was kept at 100 µL, containing 60 µL of Na_2HPO_4 buffer with a concentration of 50 mM and a pH of 7.7. Added test

compound (well-1) with volume of 10μL and a concentration of 0.5mM followed by the addition of an enzyme of 10 μL (0.005 unit well-1). Pre-incubation of substances at 37°C for 10 min was achieved. Reaction was started by addition of 10μL of 0.5mM well-1 substrate (acetylthiocholine iodide/ butyrylthiochloine chloride) followed by addition of 10μL DTNB (0.5mM well-1). Absorbance at 405nm was measured after 15 min of incubation at 37°C using the 96-well plate reader Synergy HT, BioTek, US. All experiments were done with their respective controls in triplicate. Standard drug used was Donepezil. The % inhibition was computed using the following equation

Inhibition (%) = Control – Test/control x 100

Control EZ-Fit Enzyme kinetics software (Perrella Scientific Inc. Amherst, USA) was used for the calculation of IC_{50} values.

S.3 Molecular docking protocol

The crystal structure was retrieved from the protein data bank (PDB) and further, the structure was optimized by removing the water molecules, hetero atoms, and cofactors. Hydrogen bonds, missing atoms, and charges were computed. The synthesized pyrazolone derived thiazolidinone based chalcone scaffolds used in these docking studies was prepared and optimized using built and Ligand Preparation module implemented in Discovery Studio 2018 (Dassault Systemes BIOVIA, USA). For the purpose of docking; Gold docking tool was used, Ligand preparation includes generating various tautomer's, assigning bond orders and stereochemistry. Additionally, receptor grid was generated around the AChE and BuChE active site by choosing centroid of complexed ligand (Montbretin A). The active site was defined with a radius of 12 Å around the Montbretin A binding site. Docking calculations were accomplished using Chem PLP scoring function [45].

S.3 DFT assay protocol

The geometric parameters and energies were computed by density functional theory at the B3LYP/CEP-31G level of theory, using the GAUSSIAN 98W package of the programs [46], on geometries that were optimized at CEP-31G basis set. The high basis set was chosen to detect the energies at a highly accurate level. The atomic charges were computed using the natural atomic orbital populations. The B3LYP is the key word for the hybrid functional [47], which is a linear combination of the gradient functionals proposed by Becke [48] and Lee, Yang and Parr [49], together with the Hartree-Fock local exchange function [50]. UV spectra were recorded in Rigol,

Ultra- 3000 series in Enzymology and Fungal Biotechnology Lab, Faculty of Science, Zagazig University.

44. Ellman, G. L.; Courtney, K. D.; Andres Jr, V.; & Featherstone, R. M. A new and rapid colorimetric determination of acetylcholinesterase activity. *Biochem. pharmacol.* **1961**, *7*(2), 88-95.
45. Korb, O.; Stutzle, T.; & Exner, T. E. Empirical scoring functions for advanced protein−ligand docking with PLANTS. *J.chem. inform. model.* **2009,** *49*(1), 84-96.

46. Frisch, M.J.; *Gaussian 98, Revision A.9*; Gaussian INc.: Pittsburgh, PA, USA. **1998**.
47. El-Baz, A.F.; Sorour, N.M.; Shetaia, Y.M. Trichosporon jirovecii-mediated synthesis of cadmium sulfide nanoparticles. *J. Basic Microbiol.* **2016,** *56*, 520–530.
48. Becke, A.D. Density-functional exchange-energy approximation with correct asymptotic behavior. *Phys. Rev.* **1988**, *38*, 3098–3100.
49. Lee, C.; Yang, W.; Parr, R.G.; Development of the Colle-Salvetti correlation-energy formula into a functional of the electron density. *Phys. Rev. B.* **1988**, *37*, 785–789.
50. Flurry, R.L. Jr. *Molecular Orbital Theory of Bonding in Organic Molecules*; Marcel Dekker: New York, NY, USA. **1968**.
